# Modulating swallowing-related functional connectivity and behavior *via* modified pharyngeal electrical stimulation: A functional near-infrared spectroscopy evidence

**DOI:** 10.3389/fneur.2022.1006013

**Published:** 2022-10-10

**Authors:** Xue Zhang, Hui Xie, Xiaolu Wang, Zengyong Li, Rong Song, Yilong Shan, Chao Li, Jiemei Chen, Jiena Hong, Xin Li, Guifang Wan, Yaowen Zhang, Delian An, Zulin Dou, Hongmei Wen

**Affiliations:** ^1^Department of Rehabilitation Medicine, The Third Affiliated Hospital of Sun Yat-sen University, Guangzhou, China; ^2^Beijing Key Laboratory of Rehabilitation Technical Aids for Old-Age Disability, National Research Center for Rehabilitation Technical Aids, Beijing, China; ^3^Key Laboratory for Biomechanics and Mechanobiology of the Ministry of Education, School of Biological Science and Medical Engineering, Beihang University, Beijing, China; ^4^Key Laboratory of Sensing Technology and Biomedical Instruments of Guangdong Province, School of Biomedical Engineering of Sun Yat-sen University, Guangzhou, China

**Keywords:** modified pharyngeal electrical stimulation, swallow, neuroplasticity, functional near-infrared spectroscopy, functional connectivity

## Abstract

**Introduction:**

Modified pharyngeal electrical stimulation (mPES) is a novel therapeutic modality for patients with neurogenic dysphagia. However, the underlying neural mechanism remains poorly understood. This study aimed to use functional near-infrared spectroscopy (fNIRS) to explore the influence of mPES on swallowing-related frequency-specific neural networks and ethology.

**Methods:**

Twenty-two healthy right-handed volunteers participated in the study. Each participant was randomly assigned to either the sham or the mPES group and provided a 10-min intervention program every day for 5 days. Oxyhemoglobin and deoxyhemoglobin concentration changes verified by fNIRS were recorded on days 1, 3, and 5. Five characteristic frequency signals (0.0095–2 Hz) were identified using the wavelet transform method. To calculate frequency-specific functional connectivity, wavelet phase coherence (WPCO) was adopted. Furthermore, behavioral performance was assessed pre- and post-mPES using a 150 ml-water swallowing stress test.

**Results:**

Compared with sham stimulation on day 1, the significantly decreased WPCO values were mainly associated with the dorsolateral prefrontal lobe, Broca's area, and middle temporal lobe. Compared with the sham mPES on day 1, the mPES showed a noticeable effect on the total swallow duration. Compared with the baseline, the WPCO values on days 3 and 5 showed a stepwise decrease in connectivity with the application of mPES. Furthermore, the decreased WPCO was associated with a shortened time per swallow after mPES.

**Conclusions:**

The mPES could modulate swallowing-related frequency-specific neural networks and evoke swallowing cortical processing more efficiently. This was associated with improved performance in a water swallowing stress test in healthy participants.

## Introduction

Modified pharyngeal electrical stimulation (mPES), a modified modality of pharyngeal electrical stimulation (PES), is a novel therapeutic neurostimulation tool for neurogenic dysphagia (1). The parameters for mPES include “mixing triangular and square wave, frequency: 5 Hz, pulse width: 10 ms, 10 min/day,” while the parameters of PES are “square wave, frequency: 5 Hz, pulse width: 0.2 ms, 10 min/day” (1, 2). A wide pulse duration may enhance the evoked sensory volley to the central nervous system and may lead to short- and long-term plasticity in circuits to help restore function (3–5) and reduce the fatigability of contractions (6). Meanwhile, the triangular waveform and wide pulse duration synergistically stimulate denervated muscle (7, 8). Under electromyography (EMG) guidance, mPES can elicit reflexive swallowing *via* ring electrodes located in the hypopharynx (1). The neurological mechanisms of mPES may be similar to those of PES. Previous neuroimaging studies have shown that PES can enhance the activity of the swallowing-related motor cortex (9–11). However, to the best of our knowledge, no functional near-infrared spectroscopy (fNIRS) studies have investigated the neural mechanisms of PES and mPES. Numerous studies have indicated that the swallowing-related cortical network includes the primary sensorimotor cortex, cingulate cortex, supplementary motor area, temporal lobe, inferior frontal gyrus, and inferior parietal lobule (12, 13). Nonetheless, knowledge on the effects of mPES on the functional connectivity of swallowing-related cortical networks is limited.

Functional magnetic resonance imaging (fMRI) has been used to study cortical activity during swallowing because it has nonionizing radiation, arbitrary slice scanning, multi-parameter imaging, and excellent spatial resolution (14, 15). Activation involves the prefrontal, primary sensorimotor, supplementary motor area, parietal, temporal, insula, supramarginal gyrus, and cingulate cortex (16–19). However, fMRI has several disadvantages, such as low temporal resolution, high cost, low portability, and contraindications for metal or electronic implants (20). In addition, swallowing liquid or being on command in the supine position might affect swallowing physiology and performance. Similar to fMRI, fNIRS measures the hemodynamic response or change in blood oxygenation levels driven by neural metabolic needs (21). Moreover, fNIRS is superior than fMRI in terms of high temporal resolution, portability, and low cost (21). The fNIRS can detect swallowing-related hemodynamic changes in oxygenated hemoglobin (O_2_Hb) and deoxygenated hemoglobin (HHb) concentrations in natural physiological postures. The cerebral blood oxygen signal measured using fNIRS has obvious time-frequency characteristics (22, 23). This allows analysis of not only the time-domain correlation between blood oxygen signals in various brain regions but also different frequency components corresponding to different physiological sources, which provides theoretical support for explaining the mechanism of mPES.

A method of peripheral nerve regulation could have a remote effect on the network connectivity of the entire cortical region and induce alterations in hemisphere region connectivity, and a valid mPES intervention biomarker captures the relevant neuroplasticity that contributes to behavioral change (24). Functional connectivity (FC) analysis based on fNIRS could provide new insights into the neural mechanisms underlying the reorganization of swallowing-related cortical networks induced by mPES. This study aimed to explore the influence of mPES on swallowing-related frequency-specific neural networks and ethology using fNIRS. Thus, mPES was applied to healthy participants for 5 days to observe its pre- and real-time effects on the brain networks. We hypothesized that mPES could induce neuroplastic changes in the swallowing-related cortical network, which would improve swallowing and elicit further functional network reorganization after a long-term intervention.

## Methods

### Participants

A total of 22 right-handed healthy participants (10 females, 12 males; mean age, 46.1 ± 10.7 years) participated in this study. The exclusion criteria included pregnancy; migraine; caffeinism; a history of neurological or psychiatric illness; taking any medications affecting the central nervous system; dementia; head and neck region radiation therapy; and prior surgery to the ear, nose, mouth, and throat region. The demographic characteristics and swallowing performance are shown in Table 1. Ethical approval was granted by the Ethics Committee of the Third Affiliated Hospital of Sun Yat-sen University ([2021]02-259-01). The study followed the Declaration of Helsinki and all participants provided written informed consent. Clinical trial registration: Chinese Clinical Trial Registry (ChiCTR2100054548). The execution time of this study started on 20 December 2021 and ended on 15 January 2022.

**Table 1:** The basic demographic information of the 20 participants.

**Parameters**	**mPES** **(*N* = 10)**	**Sham** **(*N* = 10)**	***P*** **-value**
Age (years)	46.1 ± 8.5	46.1 ± 12.9	0.102
Gender (male/females)	5/5	5/5	NA
Height (cm)	166.8 ± 8.2	164.0 ± 7.4	0.860
Weight (kg)	69.9 ± 13.7	67.7 ± 13.1	0.728
Education (years)	7.5 ± 2.5	6.9 ± 2.0	0.272
Handedness (right)	10	10	NA
Systolic blood pressure (mmHg)	132.2 ± 4.2	125.8 ± 5.6	0.278
Diastolic blood pressure (mmHg)	80.4 ± 2.8	71.3 ± 5.8	0.080
Number of swallows	4.6 ± 0.7	4.6 ± 0.8	0.433
Total Swallowing duration (s)	4.6 ± 1.2	4.5 ± 0.9	0.567

mPES, modified pharyngeal electrical stimulation; NA, Not applicable.

### Procedures

**Figure 1:**
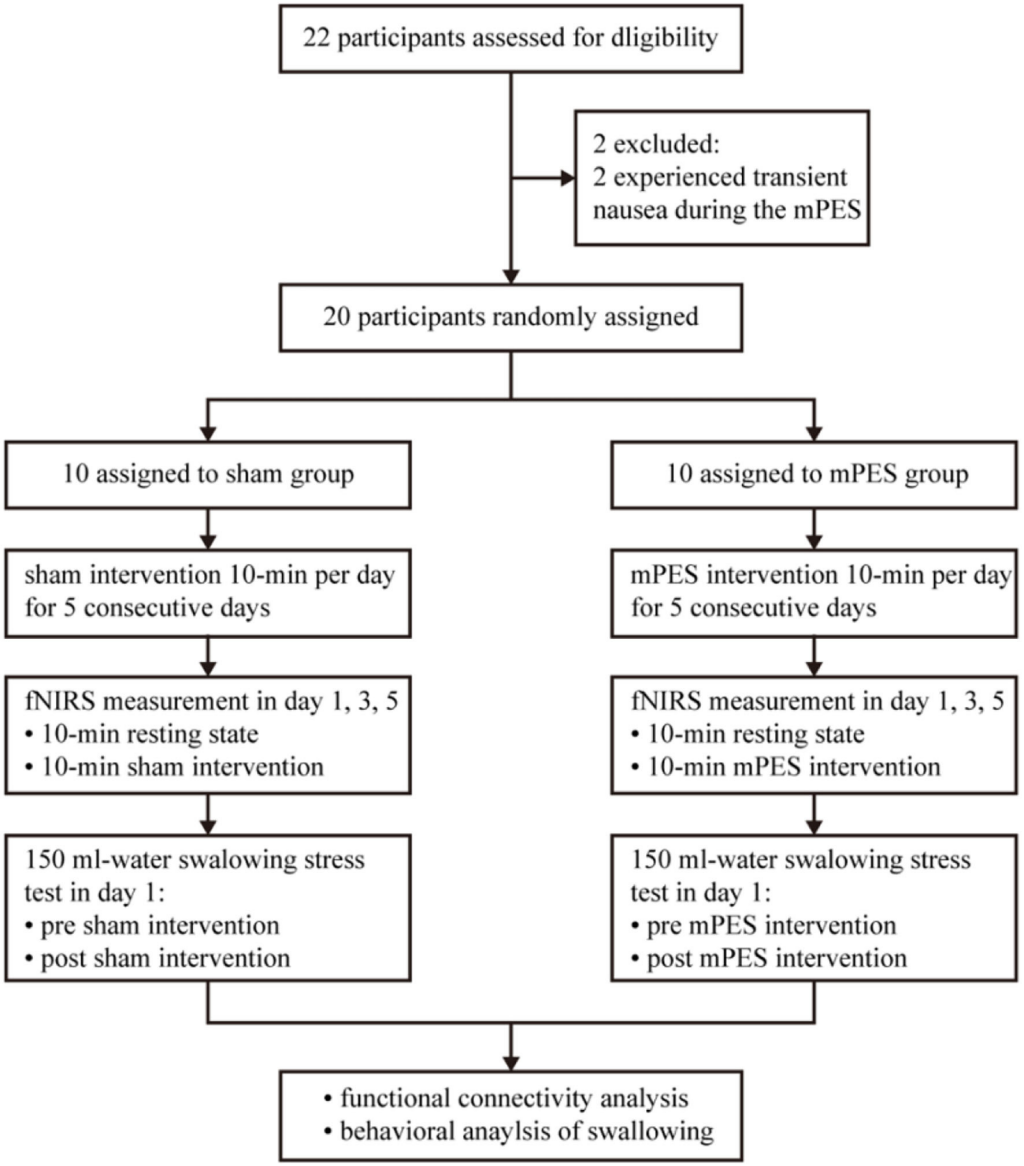
Flow diagram of the trial. mPES, modified pharyngeal electrical stimulation; fNIRS, functional near-infrared spectroscopy.

All participants were randomly assigned to either the sham or the mPES group using central computer-generated randomization numbers in sealed opaque envelopes prepared by our consultant statistician. The principal investigator WHM was responsible for allocation concealment. A qualified physiatrist ZX was responsible for performing the mPES. A research assistant XH was responsible for the fNIRS monitoring process, while a second research assistant SYL was responsible for the functional evaluation process. The data collectors and participants were blinded throughout the study period. Based on the pre-experimental results and experimental design requirements, G^*^Power 3.1.9.7 software was used for sample-size calculation of repeated measures ANOVA with parallel design. Power calculation identified a sample size of at least 20 participants to detect a 0.3 effect size with 0.8 power with significance level set at 0.05. A flowchart of the study is illustrated in Figure 1.

**Figure 2:**
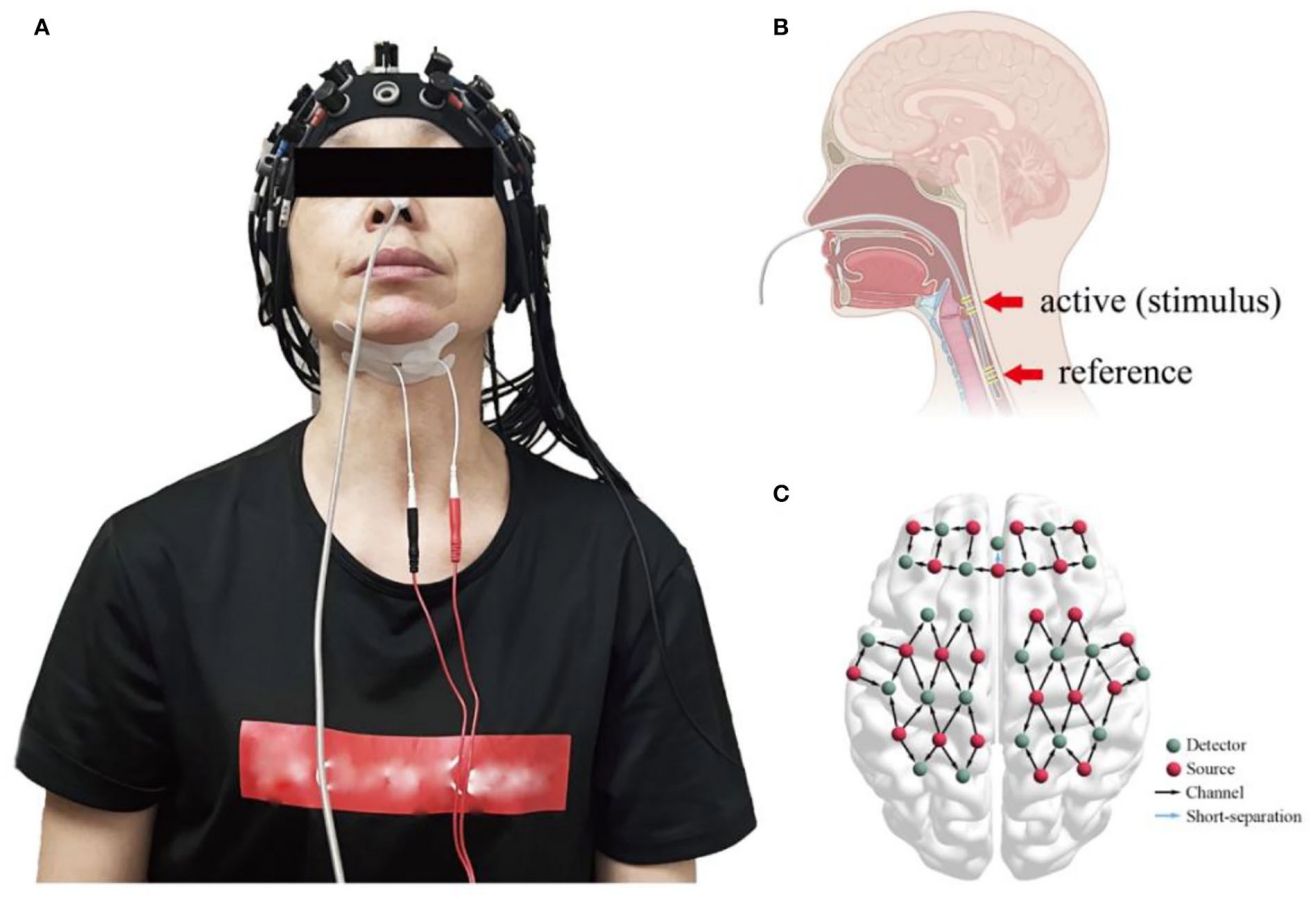
**(A)** fNIRS monitors cortical activities induced by mPES or sham procedures while bipolar skin electrodes of the surface EMG measure the duration of swallowing and the number of swallows; **(B)** the location of mPES; **(C)** cap placement and channel locations. fNIRS, functional near-infrared spectroscopy; mPES, modified pharyngeal electrical stimulation; EMG, electromyography.

Overall, prospective screening identified 22 participants were meeting inclusion criteria, and 2 of 22 participants were excluded as they experienced transient nausea during the mPES. All 20 participants performed the sham or mPES procedure for 10 min per day for 5 consecutive days, and cortical activation monitoring was carried out by fNIRS on days 1, 3, and 5. The experimental methods are illustrated in Figure 2. Behavioral performance assessments *via* a 150 ml-water swallowing stress test (25) were conducted pre-and post-mPES/sham intervention on day 1. Each participant underwent the same type of classical 150 ml-water swallow stress test and was asked to drink 150 ml of water from a disposable paper cup “as quickly as possible.” The number and duration of swallows were measured using surface EMG (ZIMMER, Neu-Ulm, Germany) (26) and the number of swallows was calculated simultaneously. The suprahyoid muscles were recorded with a pair of 10 mm diameter bipolar skin electrodes (ZIMMER, Neu-Ulm, Germany), as shown in Figure 2A.

### mPES intervention

The mPES device (1) (ZIMMER, Neu-Ulm, Germany) included a tube with two pairs of customized ring electrodes (the distance between the active electrodes for recording the EMG signals and reference electrodes was 8.8 cm), control panel for regulating parameters, and portable EMG device. The stimulus parameters were a mixed waveform (triangular and square waves) with a pulse width of 10 ms and a frequency of 5 Hz.

The electrode was placed transnasally in the pharyngeal cavity, as shown in Figure 2B. When the pharyngeal EMG fell below 20 μV for the first time, the participant was instructed to swallow the saliva. If the EMG rose rapidly above 20 μV, “the first stable below 20 μV” combined with “the rapid above 20 μV” indicated good contact between the hypopharyngeal mucosa and the active ring electrode. Thus, the position of the electrode was confirmed (1).

The mPES current intensity (CI) was detected from the initial CI (0.5 mA), and the stimulus CI was gradually increased. The perception threshold (PT) was defined as the CI at which the participant began to feel the stimulus. The maximum tolerance threshold (MTT) was the CI at which the participant felt discomfort or pain and did not want the stimulus to ramp up further. The optimal CI was calculated as *PT* + [0.75 × (*MTT* − *PT*)] (1). The operation was carried out for 10 min a day. The process of electrode location and current intensity detection was the same during mPES and sham intervention. The current was delivered during mPES, whereas, no current was delivered during the sham intervention. The electrodes were placed *via* the same nostril by the same experienced operator.

### fNIRS measurement

In the mPES and sham groups, a continuous wave fNIRS device (NirScan, Danyang Huichuang Medical Equipment Co., Ltd., China) with wavelengths of 730 nm, 808 nm, and 850 nm was used to measure cortical hemodynamic parameters (27). A total of 59 measurement channels, including 22 light source probes and 22 detector probes, were positioned symmetrically over the left and right prefrontal cortex (FP), dorsolateral prefrontal cortex (DLPFC), superior frontal cortex (SFC), premotor cortex (PMC), primary motor cortex (M1), primary somatosensory cortex (S1), middle temporal lobe (MTL), and Broca's and Wernicke's areas, as shown in Figure 2C.

### Data pre–processing

The pre-processing method of fNIRS data has been proven in previous studies (28–31). First, the 0.0095–2 Hz portion of the filtering signal was obtained using a six–order Butterworth band–pass filter to reduce non-induced components, such as low-frequency baseline drift. The changes in concentrations of O_2_Hb and HHb in each channel were calculated according to the modified Beer–Lambert law. Second, an independent component analysis was performed on the delta O_2_Hb and HHb signals of each channel to determine the components that might be related to noise and artifacts. Third, a moving average filter was used to eliminate the obvious abnormal points in the signal, and the artifact portion was removed using cubic spline interpolation. Furthermore, changes in scalp blood flow were taken from the signals using a short-separation channel with a 10 mm distance between the source and detector.

### Wavelet transform and wavelet phase coherence

The continuous wavelet transform can recognize the time-varying frequency and phase, which enables us to continuously derive the frequency content in time by adjusting the length of the wavelet windows. The Morlet wavelet can locate independent times and meet the requirements of frequency resolution, which can be used to analyze non-stationary cerebral blood oxygen signals.

The local cerebral blood oxygen concentration change detected by fNIRS is derived from spontaneous cerebral cortex activity, which reflects changes in neural activity in the cerebral cortex. The following five frequency intervals corresponding to different physiological sources have been identified by the wavelet transform (32, 33): 0.6–2 Hz, synchronization of cardiac (I); 0.145–0.6 Hz, respiratory (II); 0.052–0.145 Hz, myogenic (III); 0.021–0.052 Hz, neurogenic (IV); 0.0095–0.021 Hz, endothelial cell metabolic (V).

FC was calculated using wavelet phase coherence (WPCO), which can be used to explain the phase relation of brain function adjustments (34). The WPCO value was between 0 and 1 and could be used to quantitatively evaluate the phase coherence between two signals at a consistent degree throughout the continuous process of the time series to identify possible connectivity. The WPCO value approaching 1 indicates that there is a high degree of agreement between the two cortical regions; otherwise, the two oscillating signals are not correlated. The amplitude–adaptive Fourier transform method was applied to perform a WPCO test. A total of 50 surrogate signals with the same mean, variance, and autocorrelation functions as the original signal but with no phase correlation were produced to identify significant coherence (35, 36).

### Statistical analysis

The data were presented as mean ± standard deviation (SD). The Kolmogorov–Smirnov and Levene tests were applied to test the variance normality and homogeneity of the data at the group level. For inter-group comparison, the parameters of the resting state were taken as covariables, and covariance analysis was used to evaluate the significant differences in parameters (region-wise WPCO) between the sham and mPES states. Repeated-measures ANOVA was used to assess the significance of intragroup parameter changes in both groups, and Bonferroni correction was used for multiple comparisons. In total, there were three inter-group pairwise comparisons, and the corrected *p*-value threshold was set at *p* < 0.0167 (0.05/3). Furthermore, covariance analysis was used to evaluate behavioral data from the 150 ml-water swallowing stress test. To further verify the relationship between brain functional network connectivity and swallowing function, we computed the region-based Pearson correlation coefficients between the mPES-related WPCO changes (*WPCO* = *WPCO*_*task*_−*WPCO*_*rest*_) and EMG results in each group. The statistical significance level was set at *p* < 0.05.

## Results

The behavioral results are presented in Table 2. Compared with the sham intervention, mPES had a significant effect on total swallow duration. The stimulus current intensity of the mPES in the participants was 1–2 mA. During the first mPES intubation, only two of the participants experienced transient nausea while the remaining participants had no complaints of obvious discomfort.

**Table 2:** Behavioral results of the 150 ml-water swallowing stress test.

**Group**	**Pre (s)**	**Post (s)**	**F**	***p***
**Total swallow duration (s)**				
mPES (*N* = 10)	4.61 ± 1.19	3.81 ± 0.78	11.104	0.004
Sham (*N* = 10)	4.52 ± 0.89	4.79 ± 0.89		
**Number of swallows**				
mPES (*N* = 10)	4.60 ± 0.70	4.10 ± 0.32	3.028	0.10
Sham (*N* = 10)	4.60 ± 0.84	4.60 ± 1.07		
**Average duration per swallow**				
mPES (*N* = 10)	1.00 ± 0.21	0.93 ± 0.17	3.4	0.083
Sham (*N* = 10)	0.99 ± 0.13	1.08 ± 0.27		

mPES, modified pharyngeal electrical stimulation.

### Differences in functional connectivity

**Figure 3:**
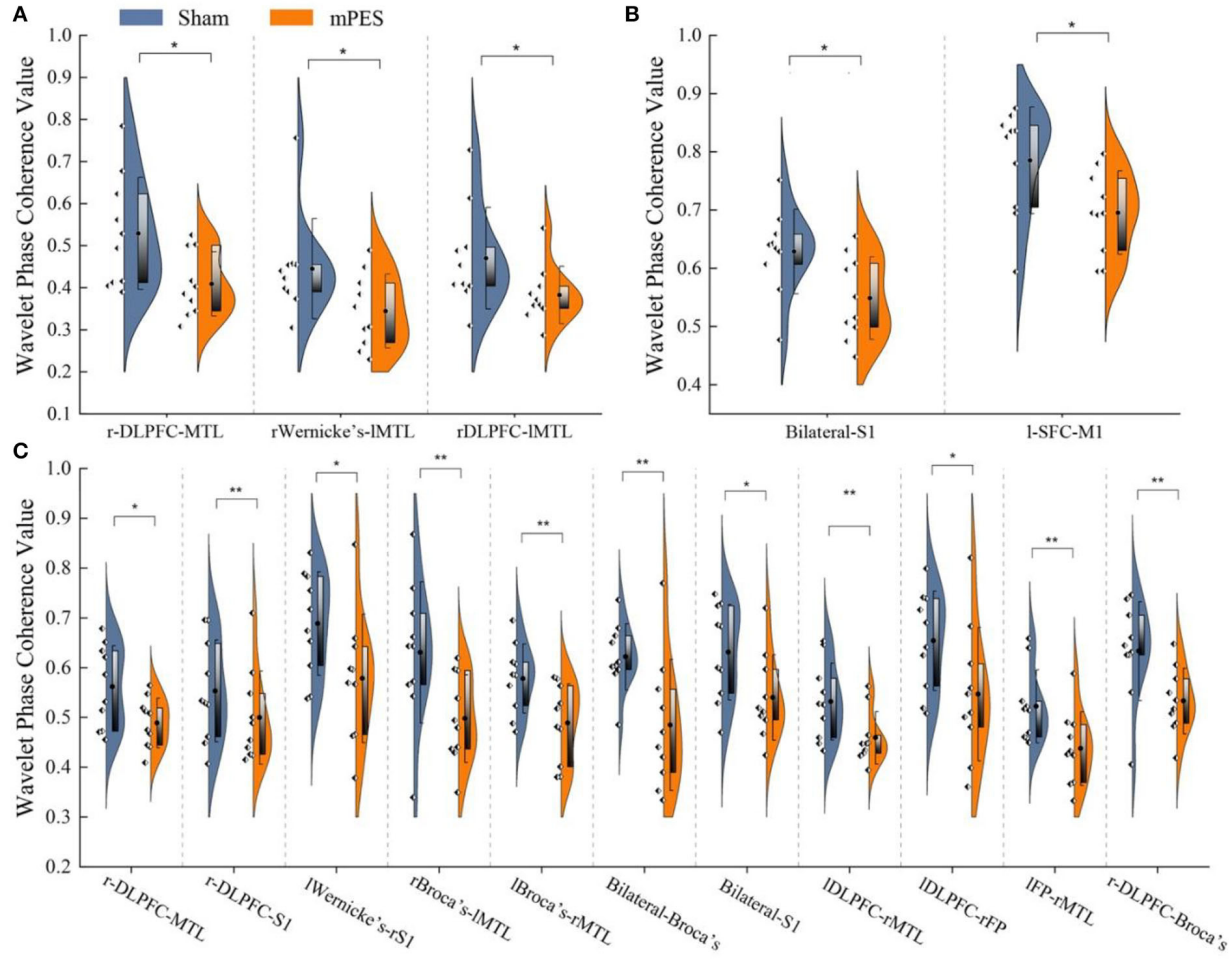
The Comparison of WPCO value between Sham and mPES group in intervals III **(A)**, IV **(B)**, and V **(C)**. WPCO, wavelet phase coherence; mPES, modified pharyngeal electrical stimulation; r, right hemisphere; l, left hemisphere; FP, frontal pole; DLPFC, dorsolateral prefrontal cortex; M1, primary motor cortex; S1, primary somatosensory cortex; MTL, middle temporal lobe; Broca's, Broca's areas; Wernicke's, Wernicke's areas. *P < 0.05, **P < 0.01.

The distribution and significant changes in WPCO values between the two groups are shown in Figure 3. In the FC network based on the WPCO values, we found that the magnitude of synchronization between the DLPFC, Broca's, and MTL in the mPES group was significantly lower than that in the sham group. Compared with the sham group, the mPES result also showed a significant lower WPCO value between r-DLPFC-MTL (*F* = 4.732, *p* = 0.044), rWernicke's-lMTL (*F* = 4.546, *p* = 0.048), and rDLPFC-lMTL (*F* = 5.059, *p* = 0.038) in interval III (Figure 3A); bilateral S1 (*F* = 4.797, *p* = 0.043) and l-SFC-M1 (*F* = 5.77, *p* = 0.028) in interval IV (Figure 3B); and bilateral Broca's (*F* = 7.692, *p* = 0.012), bilateral S1 (*F* = 4.829, *p* = 0.042), r-DLPFC-MTL (*F* = 5.278, *p* = 0.035), r-DLPFC-S1 (*F* = 6.268, *p* = 0.023), lWernicke's-rS1 (*F* = 5.645, *p* = 0.03), rBroca's-lMTL (*F* = 6.163, *p* = 0.024), lBroca's-rMTL (*F* = 8.305, *p* = 0.01), lDLPFC-rMTL (*F* = 7.402, *p* = 0.015), lDLPFC-rFP (*F* = 4.66, *p* = 0.045), lPFC-rMTL (*F* = 6.956, *p* = 0.017), and r-DLPFC-Broca's (*F* = 6.268, *p* = 0.023) in interval V (Figure 3C).

### Cumulative effects of mPES

**Figure 4:**
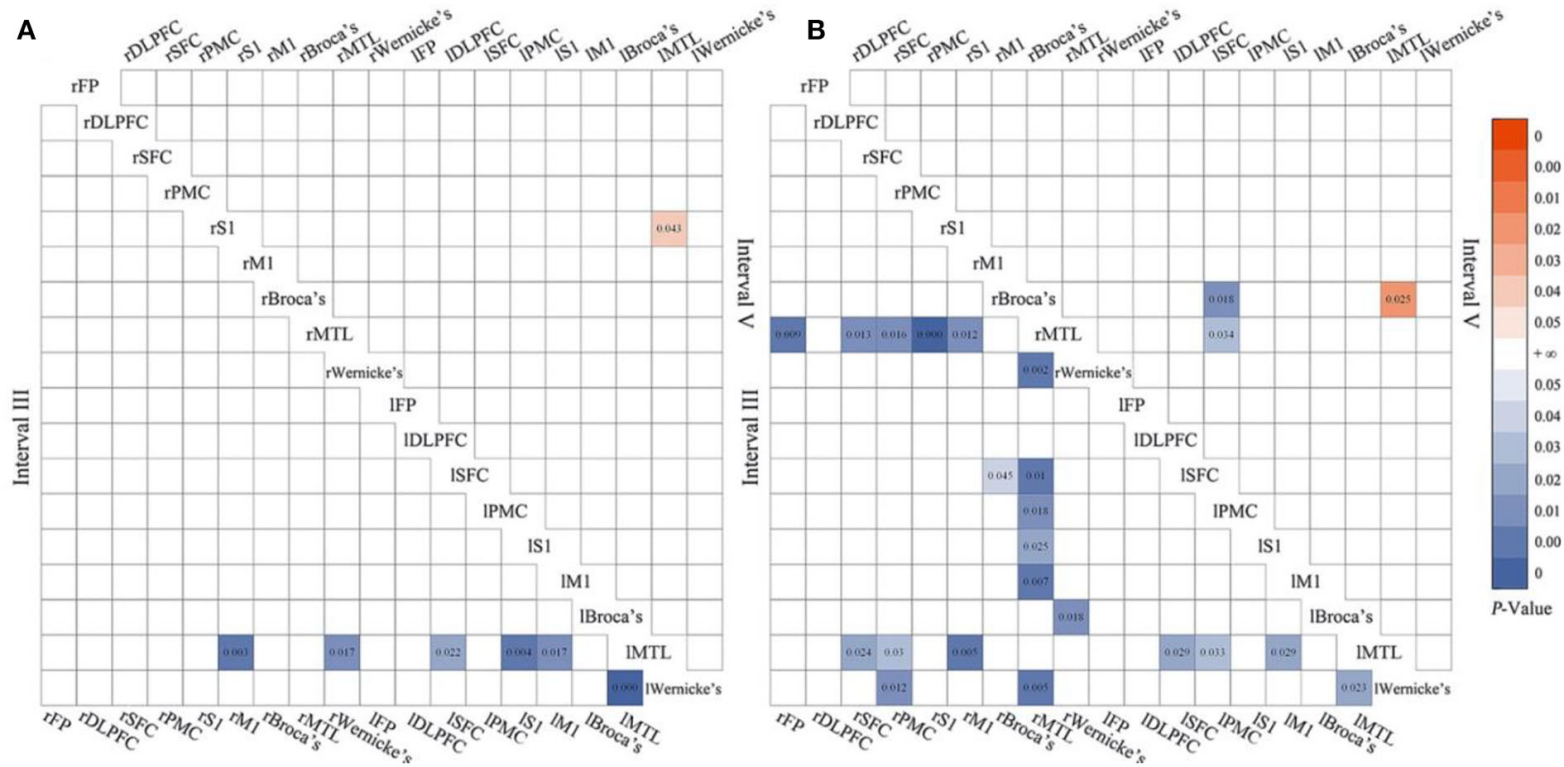
The significant changes in functional connectivity of 5 vs. 3-day mPES in intervals III and V **(A)**; the significant changes in functional connectivity of 5 vs. 1-day mPES in intervals III and V **(B)**. mPES, modified pharyngeal electrical stimulation; r, right hemisphere; l, left hemisphere; FP, frontal pole; DLPFC, dorsolateral prefrontal cortex; SFC, superior frontal cortex; PMC, premotor cortex; M1, primary motor cortex; S1, primary somatosensory cortex; MTL, middle temporal lobe; Broca's, Broca's areas; Wernicke's, Wernicke's areas.

A set of matrices showed that the statistical results of WPCO changed with the increase in the number of mPES in detail. Compared with the 3-day mPES, the WPCO value of the 5-day mPES showed a significant decrease between l-SFC-MTL (*F* = 6.224, *p* = 0.022), l-S1-MTL (*F* = 5.296, *p* = 0.004), l-M1-MTL (*F* = 0.011, *p* = 0.017), rM1-lMTL (*F* = 9.543, *p* = 0.003), rWernicke's-lMTL (*F* = 5.274, *p* = 0.017), and L-Wernicke's-MTL (*F* = 8.997, *p* < 0.001) in interval III, and a significant increase between rS1-lMTL (*F* = 5.804, *p* = 0.043) in interval V (Figure 4A). Moreover, significant changes in brain connectivity associated with MTL, Broca's, and Wernicke's occurred more extensively during the 5-day mPES than during the first intervention (Figure 4B).

### Correlations between WPCO and time per swallow

**Figure 5:**
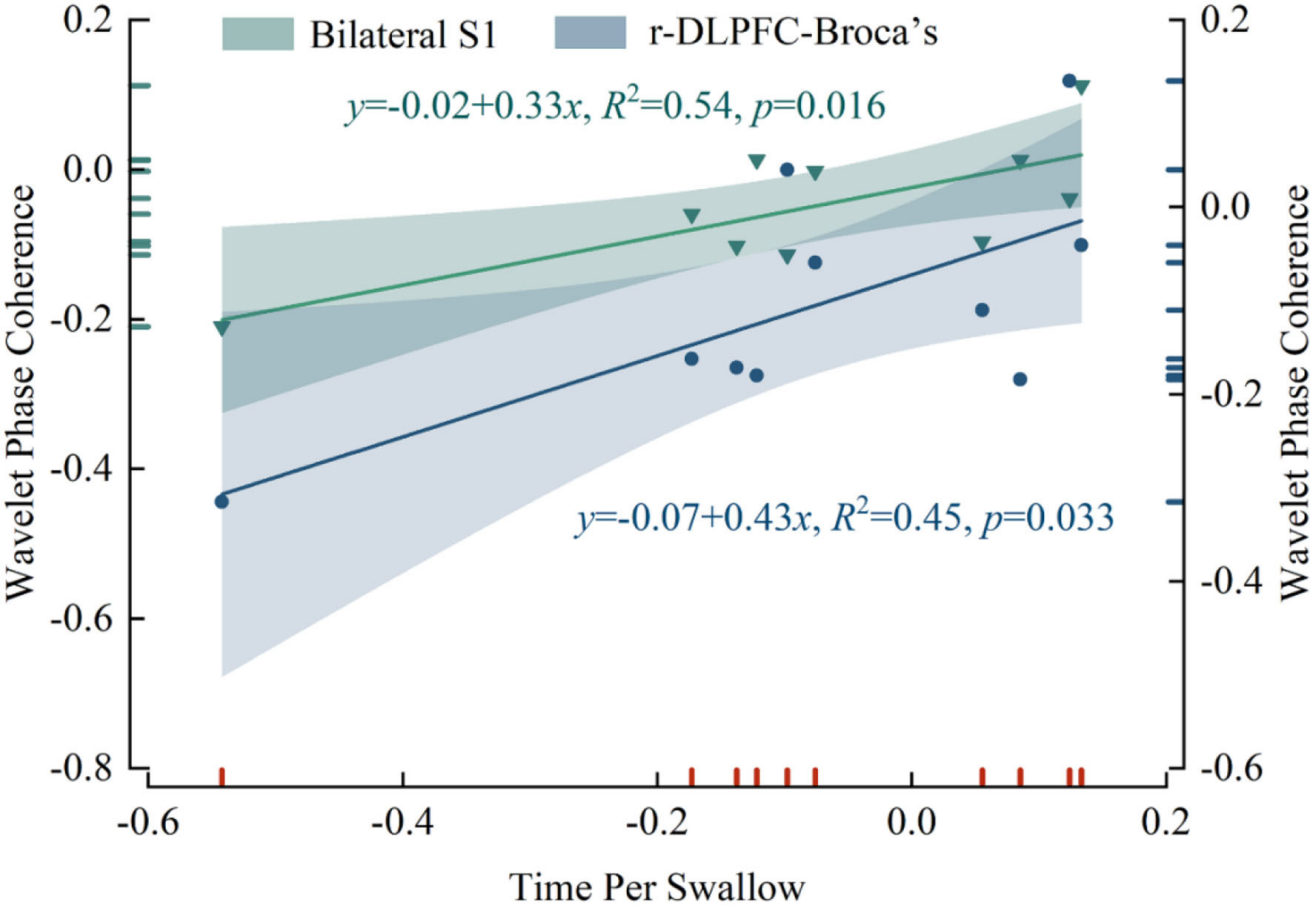
The correlation results in WPCO values and the shortened time per swallow after mPES. WPCO, wavelet phase coherence; mPES, modified pharyngeal electrical stimulation; r, right hemisphere; S1, primary somatosensory cortex; DLPFC, dorsolateral prefrontal cortex; Broca's, Broca's areas.

We examined the correlation between mPES-induced changes in WPCO values and the difference in time per swallow of post- and pre-task (Figure 5). Significant correlations were found between WPCO values and behavioral data on swallowing function. The WPCO value between the bilateral S1 (*r* = 0.733, *p* = 0.016) and r-DLPFC-Broca's (*r* = 0.674, *p* = 0.033) in the right hemisphere was positively correlated with the time per swallow. The results showed that decreased WPCO was associated with a shortened time per swallow after mPES.

## Discussion

The present study investigated the impact of mPES on frequency-specific brain functional connectivity through 5-day tracing in healthy participants using fNIRS. The seminal findings were as follows: Compared with the sham group, significantly decreased WPCO values were mainly found in the DLPFC, Broca's, and MTL in the mPES group. Relative to the baseline, the WPCO values showed a stepwise decrease in connectivity mainly associated with MTL, Broca's, and Wernicke's in intervals III and V, along with the application of mPES. WPCO values (bilateral S1 and rDLPFC-rBroca's) and shorter time per swallow after mPES were found to be significantly correlated.

FC is defined as the temporal synchronicity between spatially independent neurophysiological activities (37). It is assumed that there is a functional correlation between neurophysiological processes and time synchronization; therefore, brain regions with the same time synchronization belong to the same functional network. In this study, the effect of mPES on WPCO mainly existed at intervals III, IV, and V. The oscillations in interval III were suggested to originate locally from the intrinsic myogenic activity of smooth muscle cells in resistance vessels (38). The vascular smooth muscles contract or relax in response to an increase or decrease in intravascular pressure, respectively, and the myogenic mechanism might be partly under autonomic control. Interval IV implied that sympathetic activity and persistent autonomic nerve activity elicited vessel radii and resistance maintenance (39, 40), which provided adequate cerebral blood flow during the mPES (41, 42). In addition, the oscillations in interval V reflect the influence of endothelial-related metabolic activity during mPES (43, 44). Overall, the results of FC at intervals III, IV, and V showed that mPES could induce neuroplasticity changes in the inter- and intrahemispheric regions.

A previous neuroimaging study demonstrated an extremely stable synchronization of spontaneous activity between homologous brain regions in all bilateral cerebral hemispheres (45), which was much higher than that between non-homologous brain regions. The high correlation of neural activity between homologous regions of the bilateral cerebral hemispheres is one of the most significant characteristics of the inherent functional systems of the brain (46). We found that this inherent feature was altered in bilateral Broca's and bilateral S1 during mPES intervention. This phenomenon was also found in non-homologous brain regions, such as rWernicke's-lMTL, rDLPFC-lMTL, lWernicke's-rS1, rBroca's-lMTL, lBroca's-rMTL, lDLPFC-rMTL, lDLPFC-rPFC, and lPFC-rMTL. This finding suggests that the left and right hemispheres respond differently to swallowing tasks induced by mPES, and this conclusion was supported by previous studies (10, 47, 48). During mPES, differences in the levels of brain regions were associated with the increased asynchrony in neural activity between the bilateral hemispheres. Pharyngeal stimulation or dysfunction may be more associated with the right hemisphere.

Hemispheric specialization is an organizing principle of the human brain that has been hypothesized to contribute to fast and efficient information processing (49). In the right hemisphere, which was more influential for pharyngeal swallowing tasks, the WPCO values decreased significantly between r-DLPFC-MTL, r-DLPFC-S1, and r-DLPFC-Broca's. Even though the neural structure functions associated with swallowing are extensive, the cortical regions important for performing tasks remain unclear. According to the resource conservation theory (50, 51), the rDLPFC would deactivate for preserved mental effort during prolonged challenging task maintenance. This might explain the diminished rDLPFC in mPES-induced swallowing.

Concerning the gradually decreased brain connectivity associated with MTL, Broca's, and Wernicke's with mPES application. Although the MTL, Broca's, and Wernicke's areas have overlapping anatomy of speech and swallowing (52), they may be primarily responsible for the speech process (53). Based on the neuroplasticity of development and learning theory (54), neurons that contributed relatively little would be weakened for higher efficiency (55–57). In short, mPES could improve swallowing processing efficiency, and long-term mPES could further induce functional connectivity reorganization.

Regarding ethological behaviors, decreased functional connectivity was associated with better swallowing performance. Alterations in swallowing duration were minor but statistically significant, which could be attributed to healthy participants. Moreover, the shortened time per swallow was associated with decreased WPCO of bilateral S1 and r-DLPFC-Broca's, implying that mPES might modulate these brain regions to increase swallowing processing efficiency. As discussed above, lS1 and rDLPFC might be the major brain regions to be excluded to improve swallowing processing efficiency.

The current study has some limitations. First, fNIRS, unlike fMRI, does not provide whole-brain measurements and is limited in measuring deep cortical and subcortical regions. Second, arterial pressure oscillations occur spontaneously as Mayer waves exist in conscious participants in the vicinity of a 0.1 Hz frequency, which might affect the FC in interval III (0.052–0.145 Hz). Therefore, the interference of the Mayer waves should be considered in future studies. Last, the study included healthy participants. As studies on patients with dysphagia would be of more interest in the field of neurorehabilitation, a follow-up study including patients would be carried out.

Notwithstanding these limitations, this study provides evidence for the modulation of functional connectivity elicited by the mPES. Moreover, the mPES is relatively passive and involves minimal compliance. The method in this study could be extended to assess changes in brain activities induced by mPES in patients with dysphagia who cannot perform volitional swallowing.

Our findings suggest that mPES could induce neuroplastic changes in the swallowing-related brain network that are associated with improved swallowing behaviors. Our study provided evidence that mPES could improve swallowing processing efficiency, and long-term mPES could further induce swallowing-related functional network reorganization. Further studies investigating the effects of this tool on the brain network and swallowing function in patients with dysphagia are necessary.

## Data availability statement

The raw data supporting the conclusions of this article will be made available by the authors, without undue reservation.

## Ethics statement

The studies involving human participants were reviewed and approved by the Ethics Committee of the Third Affiliated Hospital, Sun Yat-sen University. The patients/participants provided their written informed consent to participate in this study. Written informed consent was obtained from the individual(s) for the publication of any potentially identifiable images or data included in this article.

## Author contributions

ZD and HW: conceptualization and supervision. HW: methodology. XZ and HX: formal analysis, investigation, and writing–original draft preparation. XZ, HX, ZL, XW, RS, ZD, and HW: writing–review and editing. HW, ZL, and ZD: funding acquisition. XZ, HX, XW, XL, YS, CL, JC, JH, GW, YZ, and DA: resources. All authors contributed to the study conception and design.

## Funding

This project was supported by the National Key Research and Development Project (2020YFC2004205), the National Natural Science Foundation of China (Grant Numbers 81972159 and 81672259), the Natural Science Foundation of Guangdong Province of China (Grant Numbers 2019A1515010388 and 2020A1515010881), and the Clinical Research Special Fund Project of the Third Affiliated Hospital of Sun Yat-sen University (Voyage Plan) (YHJH201909).

## Conflict of interest

The authors declare that the research was conducted in the absence of any commercial or financial relationships that could be construed as a potential conflict of interest.

## Publisher's note

All claims expressed in this article are solely those of the authors and do not necessarily represent those of their affiliated organizations, or those of the publisher, the editors and the reviewers. Any product that may be evaluated in this article, or claim that may be made by its manufacturer, is not guaranteed or endorsed by the publisher.
