# Corrigendum: Modulating swallowing-related functional connectivity and behavior *via* modified pharyngeal electrical stimulation: A functional near-infrared spectroscopy evidence

**DOI:** 10.3389/fneur.2022.1098142

**Published:** 2022-12-22

**Authors:** Xue Zhang, Hui Xie, Xiaolu Wang, Zengyong Li, Rong Song, Yilong Shan, Chao Li, Jiemei Chen, Jiena Hong, Xin Li, Guifang Wan, Yaowen Zhang, Delian An, Zulin Dou, Hongmei Wen

**Affiliations:** ^1^Department of Rehabilitation Medicine, The Third Affiliated Hospital of Sun Yat-sen University, Guangzhou, China; ^2^Beijing Key Laboratory of Rehabilitation Technical Aids for Old-Age Disability, National Research Center for Rehabilitation Technical Aids, Beijing, China; ^3^Key Laboratory for Biomechanics and Mechanobiology of the Ministry of Education, School of Biological Science and Medical Engineering, Beihang University, Beijing, China; ^4^Key Laboratory of Sensing Technology and Biomedical Instruments of Guangdong Province, School of Biomedical Engineering of Sun Yat-sen University, Guangzhou, China

**Keywords:** modified pharyngeal electrical stimulation, swallow, neuroplasticity, functional near-infrared spectroscopy, functional connectivity

In the published article, there was an error. “PES” was incorrectly written instead of “mPES”. A correction has been made to Results, Paragraph 1:

The behavioral results are presented in Table 2. Compared with the sham intervention, mPES had a significant effect on total swallow duration. The stimulus current intensity of the mPES in the participants was 1–2 mA. During the first mPES intubation, only two of the participants experienced transient nausea while the remaining participants had no complaints of obvious discomfort.

The authors apologize for this error and state that this does not change the scientific conclusions of the article in any way. The original article has been updated.

